# Differing Effects of Younger and Older Human Plasma on C2C12 Myocytes *in Vitro*

**DOI:** 10.3389/fphys.2018.00152

**Published:** 2018-02-27

**Authors:** Ifigeneia Kalampouka, Angel van Bekhoven, Bradley T. Elliott

**Affiliations:** ^1^Translational Physiology Research Group, Faculty of Science & Technology, University of Westminster, London, United Kingdom; ^2^Engineering and Applied Science, Hogeschool Rotterdam, Rotterdam, Netherlands

**Keywords:** ageing, myostatin, GDF11, plasma conditioning, muscle, frailty

## Abstract

Ageing is associated with a general reduction of physiological function and a reduction of muscle mass and strength. Endocrine factors such as myostatin, activin A, growth and differentiation factor 11 (GDF-11) and their inhibitory peptides influence muscle mass in health and disease. We hypothesised that myocytes cultured in plasma from older and younger individuals would show an ageing effect, with reduced proliferation and differentiation in older environments. C2C12 myoblasts were grown as standard and stimulated with media conditioned with 5% plasma from healthy male participants that were either younger (*n* = 6, 18–35 years of age) or older (*n* = 6, >57 years of age). Concentration of plasma myostatin (total and free), follistatin-like binding protein (FLRG), GDF-11 and activin A were quantified by ELISA. Both FLRG and activin A were elevated in older individuals (109.6 and 35.1% increase, respectively), whilst myostatin (free and total) and GDF-11 were not. Results indicated that plasma activin A and FLRG were increased in older vs. younger participants, GDF11 and myostatin did not differ. Myoblasts *in vitro* showed no difference in proliferation rate between ages, however scratch closure was greater in younger vs. older plasma stimulated myoblasts (78.2 vs. 87.2% of baseline scratch diameter, respectively). Myotube diameters were larger in cells stimulated with younger plasma than with older at 24 and 48 h, but not at 2 h. A significant negative correlation was noted between *in vivo* plasma FLRG concentration and *in vitro* myotube diameter 48 h following plasma stimulation (*r*^2^ = 0.392, *p* = 0.030). Here we show that myoblasts and myotubes cultured in media conditioned with plasma from younger or older individuals show an ageing effect, and further this effect moderately correlates with circulating FLRG concentration *in vivo*. The effect of ageing on muscle function may not be innate to the tissue, but involve a general cellular environment change. Further work is needed to examine the effect of increased FLRG concentration on muscle function in ageing populations.

## Introduction

Human ageing is associated with a general decline in physiological function from the 3rd decade onwards, including a loss of muscle mass and strength (Kallman et al., 1990). This reduction in muscle mass and function is linked with reductions of independence, reduced metabolic health, and an increase in all-cause mortality (Newman et al., 2001; Puts et al., 2005). Western society is experiencing an ageing population, with an increasing proportion of society >65 years of age, and thus an increasing need for understanding and treatment of the ageing-related loss of muscle mass and function (Bloom et al., 2015).

This loss of muscle function does not result in muscle tissue losing its plasticity and ability to respond to anabolic stimuli. It is well-established that older individuals respond adequately to resistance training with gains in muscle mass and strength (Harridge et al., 1999; Liu and Latham, 2009). The effect of ageing on total satellite cell number within muscles is unclear to date (reviewed by Snijders et al., 2009). Reduced proliferation rates are seen in satellite cells sourced from older muscle in mice (Chakravarthy et al., 2000). However, primary cell culture from biopsies of younger or older individuals do not differ in their proliferative rate (Alsharidah et al., 2013), but may show reduced fusion capacity when cultured *in vitro* (Pietrangelo et al., 2009; Brzeszczynska et al., 2018). Further, when age-dependent satellite cell proliferation capacity is seen in mice, the provision of insulin-like growth factor (IGF) is capable of off-setting this (Chakravarthy et al., 2000). These results suggest that the muscle cells *per-se* maintain functionality in ageing, but the systemic environment may instead be key.

Indeed, ageing is associated with changes in circulating factors that are known to moderate muscle mass, including the growth factors testosterone, growth hormone, and IGF (Ratkevicius et al., 2011; Sellami et al., 2017) and pro-inflammatory cytokines (Ferrucci et al., 2005; Alvarez-Rodríguez et al., 2012). The transforming growth factor beta (TGF-β) family of signalling peptides is relatively less studied. Myostatin (or growth and differentiation factor 8, GDF8) is a TGF-β peptide family member that acts as a negative regulator of muscle mass (McPherron et al., 1997). Myostatin circulates in an endocrine manner (Zimmers et al., 2002), and this endocrine compartment of myostatin correlates with muscle mass in humans across health and disease states (Gonzalez-Cadavid et al., 1998). Closely related peptide family members include activin A and growth and differentiation family member 11 (GDF11). Indeed, GDF11's amino acid similarity is ~90% relative to myostatin, despite GDF11 acting on a wider range of tissues than myostatin, including anterior posterior patterning during development, visceral organ development and neural development (Walker et al., 2016). Due in part to their structural similarity, circulating myostatin, activin A and GDF11 share inhibitory binding proteins, including follistatin, follistatin-like binding protein (FRLG) and GDF-associated serum proteins 1 and 2 (GASP-1/2; Nakamura et al., 1990; Hill et al., 2002; Amthor et al., 2004; Lee and Lee, 2013).

Plasma myostatin peptide concentration does not appear to change with age, despite older individuals showing reduced muscle mass (Ratkevicius et al., 2011). Mixed reports exist as to the changes in GDF11 with age, with one report of an increase with age (Egerman et al., 2015), whilst another reports a decrease (Sinha et al., 2014). We recently reported that chronically highly active older individuals showed increased GDF11 concentration over sedentary, aged matched controls (Elliott et al., 2017).

If the ageing process induces differences in these endocrine factors and their inhibitory binding proteins, then this may explain the loss of muscle mass and function with increasing age. Thus, the aim of this experiment was to establish the effect of cellular external environment on myocyte homeostasis *in vitro*. It was hypothesised that myocytes in an older endocrine environment would show reduced proliferative and growth response, relative to those in a younger environment.

## Materials and methods

The protocol was approved by the University of Westminster Research Ethics sub-Committee. All participants gave written informed consent in accordance with the Declaration of Helsinki prior to their participation. Participants (*n* = 12) were male, declared healthy, and equally divided into younger (*n* = 6, 18–35 years of age), or older (*n* = 6, >57 years of age. Age was recorded to the nearest month (recorded in a decimal format, e.g., 52 years 6 months = 52.5 years). Exclusion criteria including regular (daily) smoking, clinically unstable conditions, and a BMI < 18 or > 30.

Participants were requested to avoid strenuous activity for 24 h and fast for 12 h prior to the morning of testing. Participants presented at the research facility between 08:00 and 11:00 for testing. Following measurement of height and weight (287 dp, Seca, Germany) in minimal clothing, fat mass (FM), fat free mass (FFM), and skeletal muscle mass (SkM) was quantified by bioelectric impedance (mBCA 515, Seca, Germany). Participant characteristics are summarised in Table 1.

**Table 1:** Participant characteristics, grouped into younger (*n* = 6) and older (*n* = 6).

	**Younger**	**Older**	***P*-value**
Age (decimal years)	26.5 (1.5)	64.2 (2.1)	<**0.001**
Body mass index	24.3 (0.3)	26.4 (1.6)	0.217
Fat mass (kg)	15.2 (2.5)	23.4 (2.5)	**0.041**
Fat free mass (kg)	61.7 (2.2)	55.3 (1.3)	**0.032**
SkM (kg)	30.1 (1.3)	26.3 (0.8)	**0.028**
Grip strength (kg)	46.6 (2.2)	38.6 (3.9)	0.102
6MWT (m)	629.3 (27.3)	551.8 (32.5)	0.098

*BMI, Body mass index; SkM, skeletal muscle mass; 6MWT, 6 min walk test. Significant differences indicated by bolded p-values*.

Approximately 12 mL of whole blood was then collected via a convenient cubital vein in a standard manner into lithium heparin coated tubes. Collected whole blood was immediately spun (4°C, 5 000 RPM, 10 min) and resultant plasma was aliquoted and stored at −80°C for further testing.

Physical functionality of participants was assessed by hand grip dynamometry (T.K.K. 5401, Takei Scientific Instruments, Japan) and a 6 min walk test (6MWT). For grip strength, each participant stood with their dominant arm hanging straight and semi-pronated (palm facing medially). Participants were asked to give a maximal effort with verbal encouragement for 3 s, with 60 s between efforts. Grip strength was recorded three times, with the maximal value reported here. The 6MWT was performed in a standard manner (ATS Committee on Proficiency Standards for Clinical Pulmonary Function Laboratories, 2002) on a pre-marked indoor track. A hand counter was used to record distance, measured to the nearest meter.

### Enzyme-linked immunosorbent assays

Concentration of circulating myostatin within plasma (free and total, DGDF80), activin A (DAC00B), FLRG (DFLRG0) and GDF11 (DY1958) were measured by commercial ELISA (all R&D Systems, UK). Briefly, plasma samples were brought to room temperature before analysis. Total myostatin was measured by acidification of plasma samples (100 μL plasma incubated with 50 μL 1 M HCI for 10 min at room temperature, then 50 μL of 1.2M NaOH/0.5 M HEPES) and finally 200 μL calibrator diluent (RD5-26) for a 1:4 dilution factor, whilst plasma samples for free myostatin was diluted 1:4 in calibrator diluent, before resultant mix was loaded at 100 μL per well, as we describe previously (Elliott et al., 2017). Activin A, FLRG and GDF11 were measured with 100 μL of plasma loaded per well. Incubation times, conditions and washes followed manufacturer's instructions. All plates were read at 450 nm and blanked to 570 nm (FLUROstar Optima, BMG Labtech, UK). Recombinant myostatin (894410), activin A (890709), FLRG (894136), and GDF11 (844380) was used as standards, all provided by R&D (UK). All samples and standards were measured in triplicates.

### Cell culture

Myoblasts from the established C2C12 cell line were seeded (passage 11) into T75 flasks in triplicate in growth media (GM; Dulbecco's modified Eagle media with 10% fetal bovine serum, penicillin and streptomycin) in standard conditions (37°C, 5% CO_2_,100% humidity) with media changed every 48 h until ~ 80% confluent for seeding into experimental conditions. All experimental plates (described below) were seeded at an initial density of ~3.0 × 10^4^ cells.mL^−1^. All *in vitro* experiments described hereafter were performed with an *n* = 6 per group (young or old) with each individual run in triplicate.

### Cell proliferation

To assess the effect of younger or older plasma on myoblast proliferation, cells were seeded in 6 well plates in GM + 5% plasma (younger or older). Cell proliferation was assessed at 24 and 48 h. Briefly, following aspiration of media and washing with dPBS, cells were trypsinized (TrypLE, Gibco, 12605) for 5 min at 37°C to ensure complete cell removal, then resuspended. Cell counts per well was measured in duplicate, utilising an automated cell counter (Cell Countess II, Thermo Fisher, UK).

### Scratch assay

To assess the effect of younger or older plasma on myoblast migration, cells were seeded in 6 well plates in GM, and proliferated as standard until >80%. Scratch assays were performed as described by Liang et al. (2007). Briefly, cells were scratched once in a single, fluid motion using a sterile p200 tip to remove a linear section of cells. Cells were then washed with dPBS, before the addition of GM + 5% plasma (younger or older). Cells were immediately photographed (10x, 1.3 megapixel digital camera) in 3 random locations per scratch, then incubated in standard conditions for 4 h before repeating photographs. Scratch size was quantified in ImageJ (version 1.49, NIH, USA) by an individual blinded to condition and time, and closure calculated as percent of individual baseline.

### Myotube diameter

Following seeding in 6 well plates, myoblasts were grown to ~80% confluency as standard. Near confluent cells were switched to differentiation media (DM; Dulbecco's modified Eagle media with 2% horse serum, penicillin, and streptomycin), with DM changed every 24 h for 96 h. Following this, cells were stimulated with DM + 5% plasma (younger/older), and photographed immediately in 3 random locations per plate, as described above. Cells were further photographed at 4, 24, and 48 h in the same manner. Cell diameter was quantified at three different locations along the cells length, in 10 random cells per image, by an individual blinded to condition and time utilising ImageJ.

### Statistical analysis

Normality of data was checked for by Shapiro–Wilk test. Differences between younger and older characteristics, Activin A and FLRG ELISA concentrations, and scratch assay results were established by two-tailed, unpaired *t*-tests, whist effect of age on GDF11 was established by a Mann Whitney test. Concentration of total and free myostatin, cell proliferation, and myotube diameters were compared with a mixed-model two-way repeated measures ANOVA, with *post hoc* testing performed in the method of Bonferroni when indicated. Pearson's correlation was used to examine associations between endocrine factors that differed between younger and older individuals and *in vitro* results. SPSS (version 23, IBM) was used for all statistical analysis, and all figures were prepared in GraphPad Prism (version 5, GraphPad Software). Results are given as “mean [se]”, except GDF11, which is given as “median [lower–upper quartile].”

## Results

### FLRG differs between younger and older individuals

**Figure 1:**
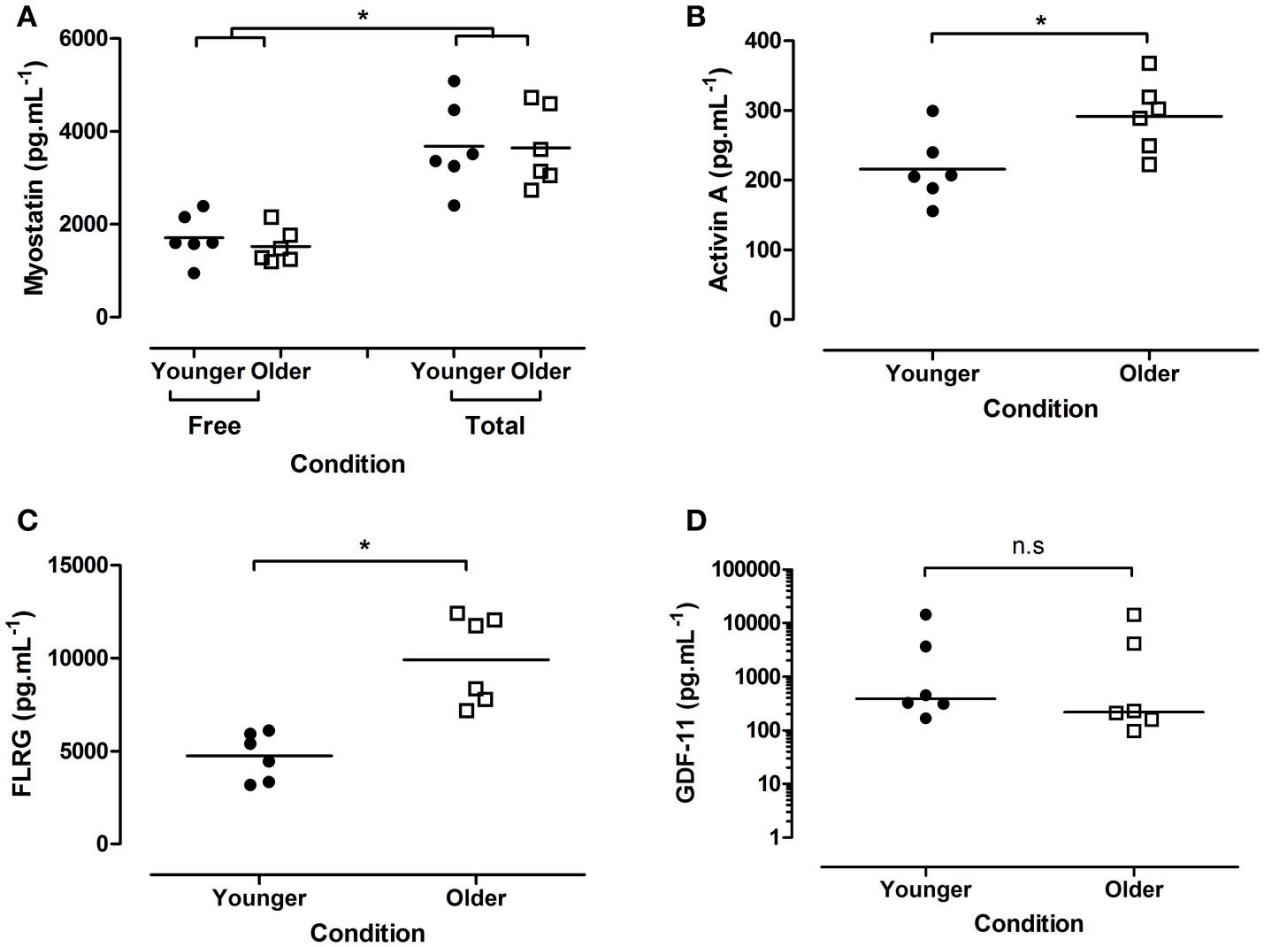
Concentration of plasma endocrine factors from younger and older participants. **(A)** Myostatin (free and total), **(B)** activin A, **(C)** FLRG, and **(D)** GDF-11 (all pg.mL^−1^). Data are presented as grouped dot plots, with horizontal lines indicated mean in panels **(A–C)**, median in panel **(D)**. Note panel **(D)** is presented on a logarithmic y axis. N = 6 per group, closed circles indicate younger, open squares older participants. ^*^Indicates significant differences as marked.

To establish the effect of age on the concentration of myostatin and myostatin-interacting peptides, plasma from younger and older individuals was used. A two-way mixed model (age × peptide state) ANOVA showed no interaction for myostatin (*p* = 0.610), nor an effect of age (*p* = 0.771). As expected, a significant effect of peptide state is seen (free 1618.9 [125.9], total 3664.1 [247.1] pg.mL^−1^; *p* < 0.001; Figure 1A).

Plasma activin A was significantly higher in older individuals (younger 215.9 [20.2], older 291.6 [21.0] pg.mL^−1^; *p* = 0.026; Figure 1B), as was plasma FLRG (younger 4731.6 [522.5], older 9915.8 [975.1] pg.mL^−1^; *p* < 0.001; Figure 1C). No difference in GDF11 was seen between groups (younger 388.4 [274.9–6339.8], older 219.4 [143.2–6694.8]; *p* = 0.394; Figure 1D).

### Cells grown in younger plasma show a protective effect

C2C12 cells were stimulated with standard GM or DM + 5% older or younger plasma for varying lengths of time before proliferation, scratch damage recovery and differentiation capacity was assessed.

**Figure 2:**
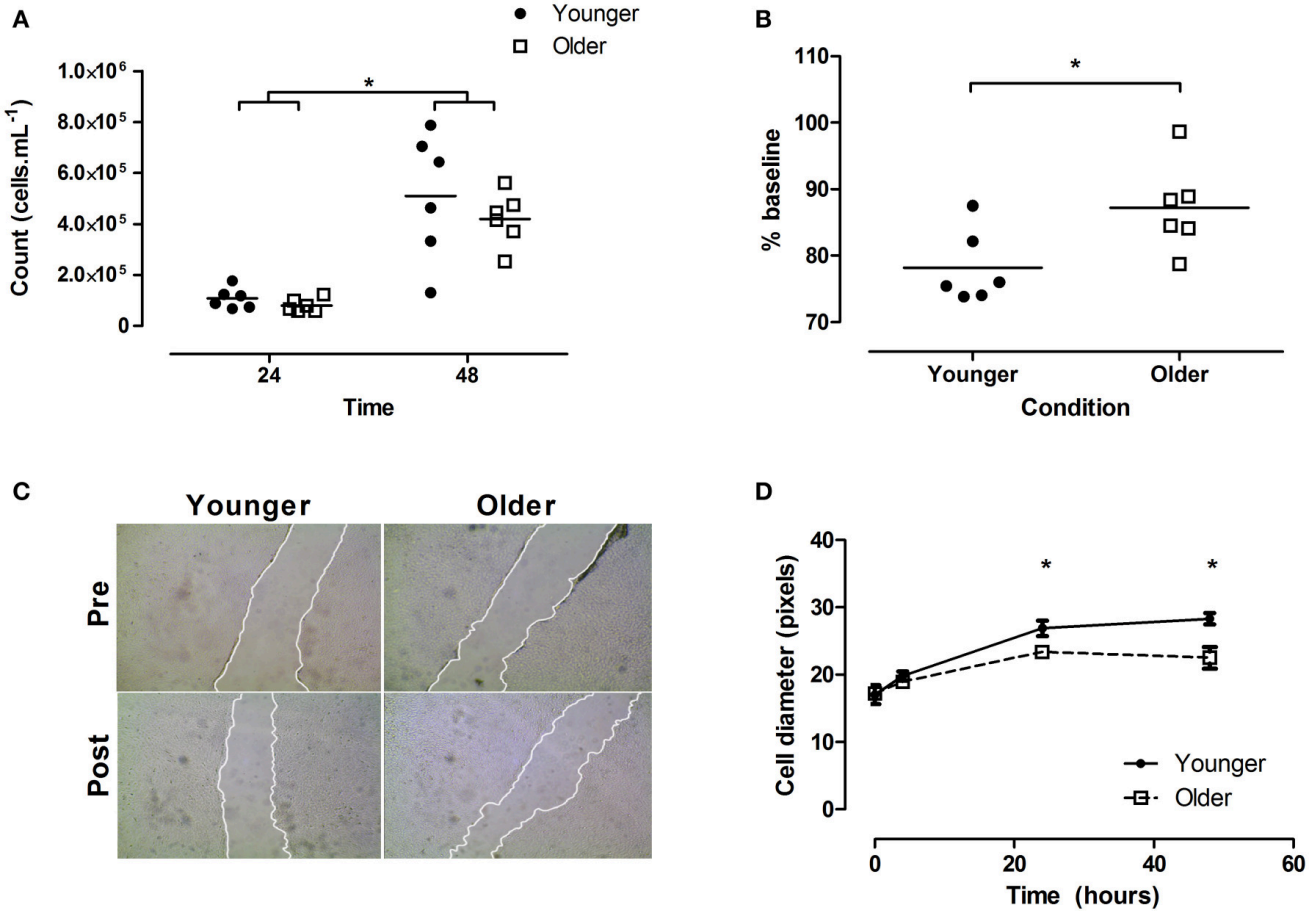
Effect of plasma conditioned media on myoblast proliferation, migration, and myotube size *in vitro*. **(A)** Proliferation rate of cells at 24 and 48 h, **(B)** scratch closure (% of baseline) 4 h post scratch, with **(C)** showing representative images. Note edges have been highlighted for publication. **(D)** myotube diameters. Horizontal lines in **(A,B)** indicate group means. Error bars in **(D)** indicate standard error. *N* = 6 per group, closed circles indicate younger, open squares older participants. ^*^Indicates significant differences as marked in **(A,B)**, significant difference between older vs. younger group at marked time-point in **(D)**. Younger/Older conditions represent cell GM **(A–C)** or DM **(D)** conditioned with 5% plasma from younger or older individuals, respectively.

No age × time interaction was seen on the proliferation capacity of cells (*p* = 0.535), nor a main effect of age (*p* = 0.367). As expected, there was a main effect of time witnessed, with significantly more cells present at 48 h, relative to 24 h (*p* < 0.001; Figure 2A).

When cells were scratched and then allowed to regenerate for 4 h, post scratch size was smaller in the younger relative to the older condition (younger 78.2 [2.2], older 87.2 [2.7]% of baseline; *p* = 0.0281; Figure 2B, representative images shown in Figure 2C).

Following replacement with conditioned media, myotube cell diameters showed a significant age × time interaction (*p* = 0.008). Specifically, *post hoc* testing suggested younger cells were larger at 24 (26.8 [0.5] vs. 23.4 [0.5] pixels; *p* < 0.05) and 48 h (28.3 [0.3] vs. 22.5 [1.6] pixels; *p* < 0.05; Figure 2D).

**Figure 3:**
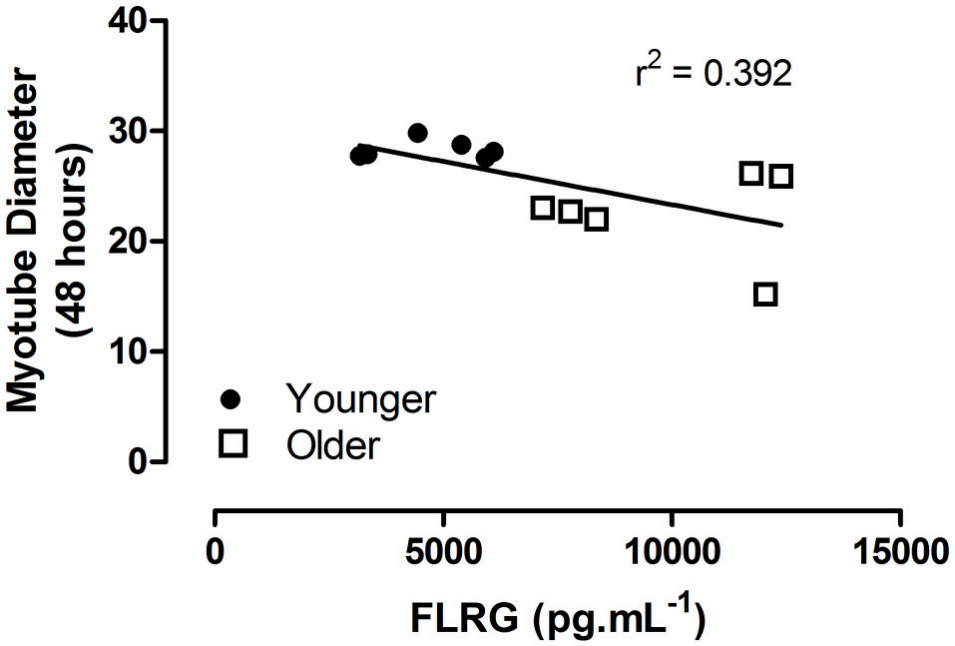
Myotube diameter at 48 h post plasma stimulation as a function of *in vivo* plasma concentration of FLRG (pg.mL^−1^). Linear regression indicated, independent of age group. Closed circles indicate younger, open squares older participants.

A significant correlation was noted between myotube diameter at 48 h and the plasma concentration of FLRG, independent of participant age (*r*^2^ = 0.392, *p* = 0.030; Figure 3), but not for activin A (*r*^2^ = 0.123, *p* = 0.264). No correlations were noted between scratch closure and FLRG or activin A.

## Discussion

In line with our hypothesis, here we demonstrate that plasma from older individuals induces both reduced myoblasts migration and a reduced myotube diameter, both relative to those stimulated with younger plasma. The primary endocrine difference reported here between younger and older individuals was in the myostatin and GDF11 inhibitor FLRG, thus it is also of interest to note that circulating FLRG concentration *in vivo* correlated with the individual myotube diameter response *in vitro*. However, no difference in GDF11, free myostatin or total myostatin plasma concentration was noted between ages.

By utilising C2C12 myoblasts as a “clean” cell line, independent of ageing effects, the effects we see from plasma conditioning of cellular media and subsequent stimulation of C2C12 cells can be deduced to be plasma-dependent. We developed this model on the observation that mixed results have been reported for an ageing effect on satellite cells and muscle functionality. Indeed, where human primary muscle cells grown *in vitro* from both younger (23–25 years of age) and older donors (67–82) do not show proliferation, fusion or myotube size differences (Alsharidah et al., 2013), primary culture from older average (78.5 years of age) show reduced myotube fusion (Pietrangelo et al., 2009). Whilst the age of older groups was similar, these samples were taken from hip replacement surgery patients, thus who are likely to have experienced extended disuse prior to biopsy collection, possibly explaining differences in results. These observations lead us to hypothesise that endocrine environment differences may underlie differences in muscle mass and function between ages, and indeed the results that we present here suggest this to be the case. However, purely endocrine factors cannot completely explain loss of muscle mass and function with ageing. Alterations in mitochondrial reactive oxygen species (Desler et al., 2011), altered mechanical stimulation of muscle satellite cells (Boers et al., 2017), alterations in physical activity (Daskalopoulou et al., 2017) and increased low level inflammation (Franceschi and Campisi, 2014) are likely to interact to impair successful ageing and the preservation of muscle mass with age.

The *in vivo* parallel to the myoblast scratch assay we report here is most likely muscular injury and recovery. In this light, it is interesting to note that older individuals show anabolic resistance to amino acid feeding (Cuthbertson et al., 2005), reduced rates of satellite cells per muscle fibre (Verdijk et al., 2007), and reduced muscle recovery following immobilisation-retraining (Suetta et al., 2009), ultimately resulting in a proposed reduced rate of injury repair and recovery (Fell and Williams, 2008) and increased intermuscular fibrosis and adipose tissue, and a reduced muscle force output (Alnaqeeb et al., 1984; Delmonico et al., 2009). Whilst speculative at this point, the endocrine mechanistic reduction in scratch-recovery in our *in vitro* older condition may in part reflect the early stages of this process.

Myotube diameter was reduced in our older condition, relative to the younger. Myostatin, GDF11 and activin A have all been demonstrated to inhibit myoblast differentiation *in vitro* (Souza et al., 2008), whilst follistatin and FLRG can be used to promote fusion, but indirectly via myostatin-dependent myotube inhibition (Hill et al., 2002; Iezzi et al., 2004). With no difference in myostatin or GDF11, we would suggest that the either increased activin A concentration has resulted in reduced fusion in the older group, or the increase in FLRG in the older group is impairing myoblast fusion, as implied by the moderate correlation we report between myotube size and FLRG concentration. This second hypothesis seems initially unlikely, as FRLG has been demonstrated to bind and inhibit myostatin *in vivo* (Hill et al., 2002), in a similar manner to the closely related follistatin (Amthor et al., 2004), which should be expected to increase myotube size, not decrease as we report here. To the best of our knowledge, the direct effect of FLRG on myoblast proliferation and differentiation has not been directly examined *in vitro*, however it is interesting to note that FLRG does directly inhibit osteoclast fusion and differentiation (Bartholin et al., 2005).

Whilst not a primary aim of this *in vitro* experiment, is it noteworthy to report that both total and free myostatin appear to circulate at similar concentrations between younger and older individuals in the limited sample size collected here, despite the later having significantly reduced skeletal muscle mass. Similar results have been reported previously for plasma total myostatin in younger healthy males vs. older sarcopenic males (Ratkevicius et al., 2011), however we further these results with the addition of free myostatin. The same paper reports a trend toward a difference in FLRG concentration between younger and older individuals, whist both Bergen et al. (2015) and our results presented here suggest FLRG is increased in older individuals. The correlative relationship that we report here between circulating FLRG *in vivo* and myotube diameter *in vitro* is suggestive of a physiological relationship between the two.

Much recent attention has been placed on ageing and GDF11, with reports that GDF11 decreases with age in mice, and restoration of “younger” levels ameliorates age-related declines in muscular function (Sinha et al., 2014). Further, highly active older individuals show increased levels of GDF11 over inactive older individuals (Elliott et al., 2017). Whilst we report no difference between younger and older individuals here in both myostatin and GDF11, we do acknowledge the study was not powered for this outcome. However, it is of interest to note that the age groups do have similar concentration ranges of these peptides, despite the significant difference in muscle mass seen. It is tempting to infer a sensitivity to the effects of circulating myostatin/GDF11 may be seen in older individuals, helping explain this apparent contradiction, however further work would be needed to state this. Other TGF-β family members have been suggested to contribute to human ageing, and here our results show an increase in activin A, confirming prior findings (Baccarelli et al., 2001).

Whilst not measured here, it should be acknowledged that a large number of endocrine factors are suggested to change with human ageing. These include classic anabolic factors such as growth hormone, insulin and IGF (Ratkevicius et al., 2011; Sellami et al., 2017), as well as the likely catabolic, low level of pro-inflammatory cytokines such as tumour necrosis factor alpha and interleukin 1 beta (Ferrucci et al., 2005; Alvarez-Rodríguez et al., 2012). Further, more recently identified factors such as Klotho act in an endocrine manner in mice and appear to regulate lifespan (Kurosu et al., 2005). A complete picture of endocrinology of aging may need to consider these factors.

## Conclusions

In conclusion, here we demonstrate that plasma from older individuals both inhibits myoblast migration and reduces myotube diameter, relative to younger plasma. These effects appear independent of circulating myostatin and GDF11, however may involve activin-family peptide inhibitor FLRG. Whilst our results show part of ageing-related changes in muscle mass may be endocrine in nature, the interaction between this observation and other aspects of ageing need to be further explored. Further work is needed to examine the effect of increased FLRG concentration on muscle function in ageing populations.

## Author contributions

IK and BE: conceived this experiment; IK and AvB: conducted data collection and analysis; IK: wrote the first draft of this manuscript. All authors contributed to manuscript revision, read, and approved the submitted version.

### Conflict of interest statement

The authors declare that the research was conducted in the absence of any commercial or financial relationships that could be construed as a potential conflict of interest.
